# Spatial analysis and optimization of self-pickup points of a new retail model in the Post-Epidemic Era: the case of Community-Group-Buying in Xi’an City

**DOI:** 10.1007/s43762-023-00089-8

**Published:** 2023-03-20

**Authors:** Zhe Lin, Gang Li, Muhammad Sajid Mehmood, Qifan Nie, Ziwan Zheng

**Affiliations:** 1 Sino-Danish College, University of Chinese Academy of Sciences, Beijing, 100049 People’s Republic of China; 2 Economics and Business Administration, Aalborg University, Aalborg, 999017 Denmark; 3 College of Urban and Environmental Sciences, Northwest University, Xi’an, 710127 People’s Republic of China; 4 Shaanxi Key Laboratory of Earth Surface System and Environmental Carrying Capacity, Northwest University, Xi’an, 710127 People’s Republic of China; 5 Hangzhou POLYFUL Advanced Material Co., 310000 Hangzhou, People’s Republic of China; 6 School of Big-Data and Network Security, Zhejiang Police College, Hangzhou, 310053 People’s Republic of China

**Keywords:** Location optimization, CGBPs, Spatial analysis, Community life, Xi’an

## Abstract

The Community-Group-Buying Points (CGBPs) flourished during COVID-19, safeguarding the daily lives of community residents in community lockdowns, and continuing to serve as a popular daily shopping channel in the Post-Epidemic Era with its advantages of low price, convenience and neighborhood trust. These CGBPs are allocated on location preferences however spatial distribution is not equal. Therefore, in this study, we used point of interest (POI) data of 2,433 CGBPs to analyze spatial distribution, operation mode and accessibility of CGBPs in Xi’an city, China as well as proposed the location optimization model. The results showed that the CGBPs were spatially distributed as clusters at α = 0.01 (*Moran’s I* = 0.44). The CGBPs operation mode was divided into preparation, marketing, transportation, and self-pickup. Further CGBPs were mainly operating in the form of joint ventures, and the relying targets presented the characteristic of ‘convenience store-based and multi-type coexistence’. Influenced by urban planning, land use, and cultural relics protection regulations, they showed an elliptic distribution pattern with a small oblateness, and the density showed a low–high-low circular distribution pattern from the Palace of Tang Dynasty outwards. Furthermore, the number of communities, population density, GDP, and housing type were important driving factors of the spatial pattern of CGBPs. Finally, to maximize attendance, it was suggested to add 248 new CGBPs, retain 394 existing CGBPs, and replace the remaining CGBPs with farmers’ markets, mobile vendors, and supermarkets. The findings of this study would be beneficial to CGB companies in increasing the efficiency of self-pick-up facilities, to city planners in improving urban community-life cycle planning, and to policymakers in formulating relevant policies to balance the interests of stakeholders: CGB enterprises, residents, and vendors.

## Introduction

The outbreak of COVID-19 has posed a huge threat to people’s lives. In the absence of a vaccine, social distancing and total lockdown are the main measures to ease and flatten the epidemic curve (Lauer et al., 2020; Musinguzi & Asamoah, 2020). The question of how to provide basic life necessities for residents when the community is closed has become a problem that many governments must consider. A new way of online shopping and the newest form of new retail, Community Group Buying (CGB), provides a solution by setting up self-pick-up points around the community.

CGB was time for the first introduced in China in September 2016. It adopts the mode of “online booking plus offline self-pickup”. Based on the relationships of offline physical communities and online social networks, group leaders promote commodities, mainly fresh products at low prices, to community residents on social media platforms (Li et al., 2020). The next day, consumers must pick up their commodities at their designated CGBPs with the help of the group leader. In early 2020, the home quarantine and lockdown rule restricted long-distance movement of people, which accelerated the transformation process from traditional retail to CGB (Liang, 2017). As the number of consumers grows, the number of CGBPs has exploded, which brings convenience to residents, but also causes a series of social problems such as waste of resources and damage to vendors’ interests. Therefore, how to adjust and optimize the location of CGBPs, the end link of CGB logistics, is an urgent problem that needs to be solved.

In recent years, most authors have considered CGB as one of the ways of traditional online shopping and focused on its stock and supply (Cao, 2021; Liu, 2019, 2020; Yi & Zhang, 2020), marketing and business model (Zheng & Liu, 2020; Geng & Sha, 2020; Wang, 2019) and collection and delivery (Tan & Wu, 2019; Zhang, 2014). These studies covered major links in the supply chain, from production to consumption, from the perspective of economics, management, and sociology, but little attention was paid to the final self-pickup section, especially the self-pickup points. The essential difference between CGB and other online retail businesses was not highlighted. To compensate for this deficiency and provide a solution to the logistics “last mile” delivery problem (Boysen et al., 2021; Iwan et al., 2016), this study focused on offline CGBPs, which played an important role at the end of the supply chain.

CGBPs could be considered points of interest (POI), which was widely used in the spatial layout of public facilities (Qi et al., 2018; Li et al., 2019a, b; Liu et al., 2020), regional spatial range identification (Kang et al., 2018; Wang et al., 2018), and spatial relationship of urban sites (Guo et al., 2015). In recent years, it has played an important role in research on business and logistics networks, including the catering industry (Kimes, 2011; Thornton et al., 2016), traditional retail industry (Applebaum, 1966), entertainment facilities (Tang et al., 2018) and emerging collection and delivery points represented by the “Cainiao Station” and the “China Post” (Li et al., 2018, 2019a, b; Liu et al., 2019; Mehmood et al., 2022). Although previous research has identified some urban facilities’ location preferences, there has been no research assessing the spatial pattern of self-pickup facilities of CGB, focusing on spatial analysis and location optimization. To expand related research, this study examines the spatial distribution of 2,433 CGBPs (taking “XingShengYouXuan”, a CGB brand, as an example) in Xi’an, China.

The research is divided into six sections. The second part explains the study area, data source, spatial analysis methods, and the optimization model used in this paper. The third part summarizes the operation model of CGB and the basic characteristics of its relying targets (CGBPs). The fourth part shows the spatial pattern of CGBPs and examines their potential influencing factors. The fifth part evaluates the accessibility and service coverage of CGBPs based on the “community life circle” theory, by distance and time, respectively, and optimizes the location of CGBPs by combining the minimize facilities model and the maximum attendance model. Finally, this paper presents some policies and suggestions for the sustainable development of CGB, seeking to achieve both fairness and efficiency.

## Data collection and methods

### Study area

**Fig. 1:**
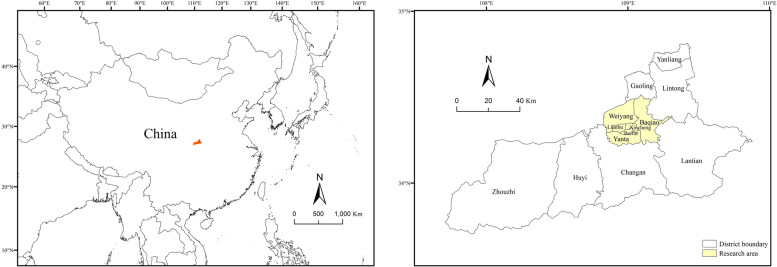
Study Area

Xi’an is the capital of Shaanxi Province and a large city located in the middle of the Guanzhong Plain in western China. It is the central city of the urban agglomeration in Guanzhong Plain and one of the nine Central National Cities of China, leading the economic development of western China. As illustrated in Fig. 1, by the end of 2021, Xi’an comprises 11 administrative districts, including 6 central districts (Beilin, Yanta, Lianhu, Weiyang, Xincheng and Baqiao), 5 suburbs (Chang’an, Yanliang, Huyi, Lintong and Gaoling) and 2 counties (Lantian and Zhouzhi), with a total area of 10,752 square kilometers and a population of 10,203,500 people, and an urbanization rate of 74.61% (Xi’an Statistics Bureau, 2020). 6 central districts with high population density and developed retail industries were selected as the study area.

CGB entered Xi’an in October 2018, so it has been developing for nearly 4 years so far. The number and location of CGBPs have become stable, providing reliable data for this study. Moreover, the area north of Qinling Mountains in Xi’an is dominated by plains, so the terrain is flat, which means that the location of CGBPs is less disturbed by terrain factors and is mostly driven by social and economic factors. Therefore, the results of the research are important for the location planning of artificial CGBPs.

### Data sources

Considering that the self-pick-up points of different CGB brands overlapped in space, “XingShengYouXuan”, a CGB brand with the longest history, the largest number of CGBPs, the largest market share and the most widely distributed, was selected as a representative of CGB. Its POI data was collected via the APP platform ‘XingShengYouXuan’ using Python. Each POI data point includes a name, address, longitude, latitude, business status, and links to store photos. After data cleaning, the valid data for open CGBPs was 2,433 items as of September 2022.

Data on the communities in Xi’an were collected from the Shaanxi Department of Civil Affairs (http://zwfw.saxmz.gov.cn/). Name, address, housing type, average housing price, etc. were all included in each data entry. After converting address information to latitude and longitude coordinates, the reliable POI data for communities was 2,760. Road network data and related POI data (including restaurants, barbershops, hotels, etc.) were obtained from Open Street Map. The Xi’an boundary data was collected from the China National Geographic Information Center (2022), while the population and GDP data was obtained from the Resource and Environment Science and Data Center (https://www.resdc.cn/Default.aspx).

### Methodology

#### Spatial correlation index

The relationship between population distribution density at the global population attributes was calculated by *Moran’s I* (Anselin & Getis, 1992; Ord & Getis, 1995, 2001). The Moran I index was used in this study to calculate the degree of aggregation of CGBPs. CGBPs show clusters when Moran’s *I* > 0 at a significant level. If Moran’s *I* = 0, the CGBPs in the study area are randomly distributed.

#### Kernel density estimation

Kernel density estimation is used to calculate the unit density of point and line feature within the specified neighborhood, and help identify hotspots where events occur most frequently (Wu, et al., 2018). The kernel density was used to identify the spatial hotspots of the CGBPs distribution in this research.

#### Standard deviational ellipse

The standard ellipse is a classic spatial distribution analysis algorithm that examines the characteristics of a point data set. The long axis of the ellipse represents the maximum diffusion direction of the points, whereas the area represents the concentration of discrete points (Newman, 1939). This study output the ellipse of CGBPs in the study area to explore their distribution direction.

#### Geodetector

The Geodetector method is a statistical tool to measure spatial stratified heterogeneity (SSH) and to make attribution for/by SSH (Liu & Yang, 2012; Zhu et al., 2015). Both factor and interaction detectors were used in our study to explore explanatory power of potential influencing factors on the CGBPs’ spatial distribution before and after the interaction.

1 $$q=1-\frac{1}{n{\sigma }^{2}}\sum\nolimits_{h=1}^{L}{n}_{h}{\sigma }_{h}^{2}$$

where $n$ is the sample size; $h = 1, 2,\cdots , L$ is the number of zones (categories) of factor X; ${n}_{h}$ is the number of samples in a zone (category) $h$; ${\sigma }_{h}^{2} and {\sigma }^{2}$ are the variance of Y within zone (category) $h$ and the total variance of Y in the study area, respectively. $q$ represents the explanatory power of a certain factor X over Y  (Jiang et al., 2021).

Interaction detection is used to test the effect of any two factors after the interaction. First, the q-value of two factors $X1$ and $X2$ on $Y$ are calculated respectively: $q(X1)$ and $q(X2)$; Then, overlay layers $X1$ and $X2$ to form a new polygon distribution, calculating the new q-value of the polygon: $q(X1\cap X2)$. Finally, compare $q(X1)$, $q (X2)$ with $q(X1\cap X2)$. The results can be divided into five categories (Table 1) (Wang & Xu, 2017).

**Table 1:** Interaction detector

Example	Criteria	Interaction effect
	$q(X1\cap X2)< Min[ q(X1) , q(X2) ]$	Nonlinear weakened
	$Min\left[ q\left(X1\right), q\left(X2\right)\right]< q\left(X1\cap X2\right)< Max\left[ q\left(X1\right), q\left(X2\right)\right]$	Single-factor nonlinear weakened
	$q(X1\cap X2) > Max[ q(X1) , q(X2) ]$	Double-factor enhanced
	$q(X1\cap X2) = q(X1) + q(X2)$	Independent
	$q(X1\cap X2) > q(X1) + q(X2)$	Nonlinear enhanced

Blue circle: $Min[ q(X1) , q(X2) ]$; Green circle: $Max[ q(X1) , q(X2) ]$; Yellow circle: $q(X1) + q(X2)$; Red inverted triangle: $q(X1\cap X2)$

#### Buffer analysis

A buffer is a zone drawn around any point, line, or polygon that encompasses all areas within a specified distance of the feature. In this study, to assess the accessibility of CGBPs by straight-line distance, point buffers were created with CGBPs as centers and 300, 500 and 1000 m as radius, corresponding to residential areas of the 5-min, 10-min and 15-min community life circle in the distance, respectively.

#### Network analysis

To evaluate the accessibility to CGBP, the network service areas, regions derived from a specific location considering all reachable roads within a given impedance (5-, 10- and 15-min), were identified, corresponding to the residential areas of the 5-, 10- and 15-min community life circle in time, respectively.

The maximize attendance model is efficiency-oriented (Church & ReVelle, 1974), which aims to make sure as much demand weight as possible is allocated to facilities, mainly for alternative facilities such as general retailers, restaurants, and fitness centers (Minghua, 2005; Hakimi, 1965; Toregas et al., 1971). The conditions for the model are as follows:

1. All CGBPs have the same attractiveness and capacity, so the number of consumers is only related to distance. The probability of residents choosing a CGBP decays according to the $N$ th power (N = 2) of the distance between the community and the CGBPs.
2. The locations of existing CGBPs, alternative CGBPs and demand points (communities) are already known.
3. All CGBPs and communities are located along the road, and the number of residents in each community is equal. $I$ represent the collection of communities (i ∈ I, i = 1, 2, 3, 4, …, m), and $J$ represents the collection of CGBPs (j ∈ J, j = 1, 2, 3, …, n).
4. ${P}_{ij}$ is the possibility for the residents living in community *i* to pick up at the CGBP *j*. ${d}_{ij}$ is the distance between the CGBP *j* and the community *i*, calculated with the actual path distance. $v$ is the speed of walking and $t$ is the maximum time for a walk commute (t = 5). Communities outside the impedance cutoff (t = 5) of all CGBPs are not allocated to any CGBP.
5. ${b}_{ij}$ represents attribution relationship. ${b}_{ij}$=1 indicates attribution, while ${b}_{ij}$=0 indicates no attribution. A community covered by more than one CGBP within the impedance cutoff is allocated only to the nearest CGBP.

The optimal location selection model is constructed as follows.

2 $$\mathrm{max}\sum\nolimits_{j=1}^{n}\sum\nolimits_{i=1}^{m}{b}_{ij}{P}_{ij}$$

3 $$\begin{array}{cc}\mathrm{s}.\mathrm{t}.& 0<\frac{{d}_{ij}}{\nu }{b}_{ij}\le t\end{array}$$

4 $$\sum\nolimits_{i\in I}{b}_{ij}\le m \forall j\in J$$

5 $${P}_{ij}=f\left({d}_{ij}^{N}\right)$$

6 $${b}_{ij}=\left\{\begin{array}{c}0,{d}_{ij}>{d}_{\left(i+1\right)j}\\ 1,{d}_{ij}\le {d}_{\left(i+1\right)j}\end{array}\right.$$

The ArcGIS location assignment tool was applied to run this model.

## Operation model and CGB-relying targets

### CGB operation model

Community group buying (CGB) is a kind of service based on physical communities offline, so the demand side drives the supply side. On the demand side, CGB companies choose group leaders, mainly community residents or store owners, who community residents trust. Similarly, their residence or store are set up as distribution nodes as well as CGBPs. Group leaders will publish commodity information from CGB companies in WeChat groups (created through their social network) for presale. Group members can place an order in WeChat groups or on the APP at low prices and designate the nearest CGBP. WeChat pay, Alipay, and other digital payment platforms can all be used to pay.

On the supply side, after the consumer completes payment, CGB companies are responsible for the collection of consumer orders, and delivery to each CGBP. Usually, commodities will arrive at each distribution node (CGBP) the next day before 4 pm., then consumers need to pick up their commodity themselves at their designated CGBP with the help of the group leader.

**Fig. 2:**
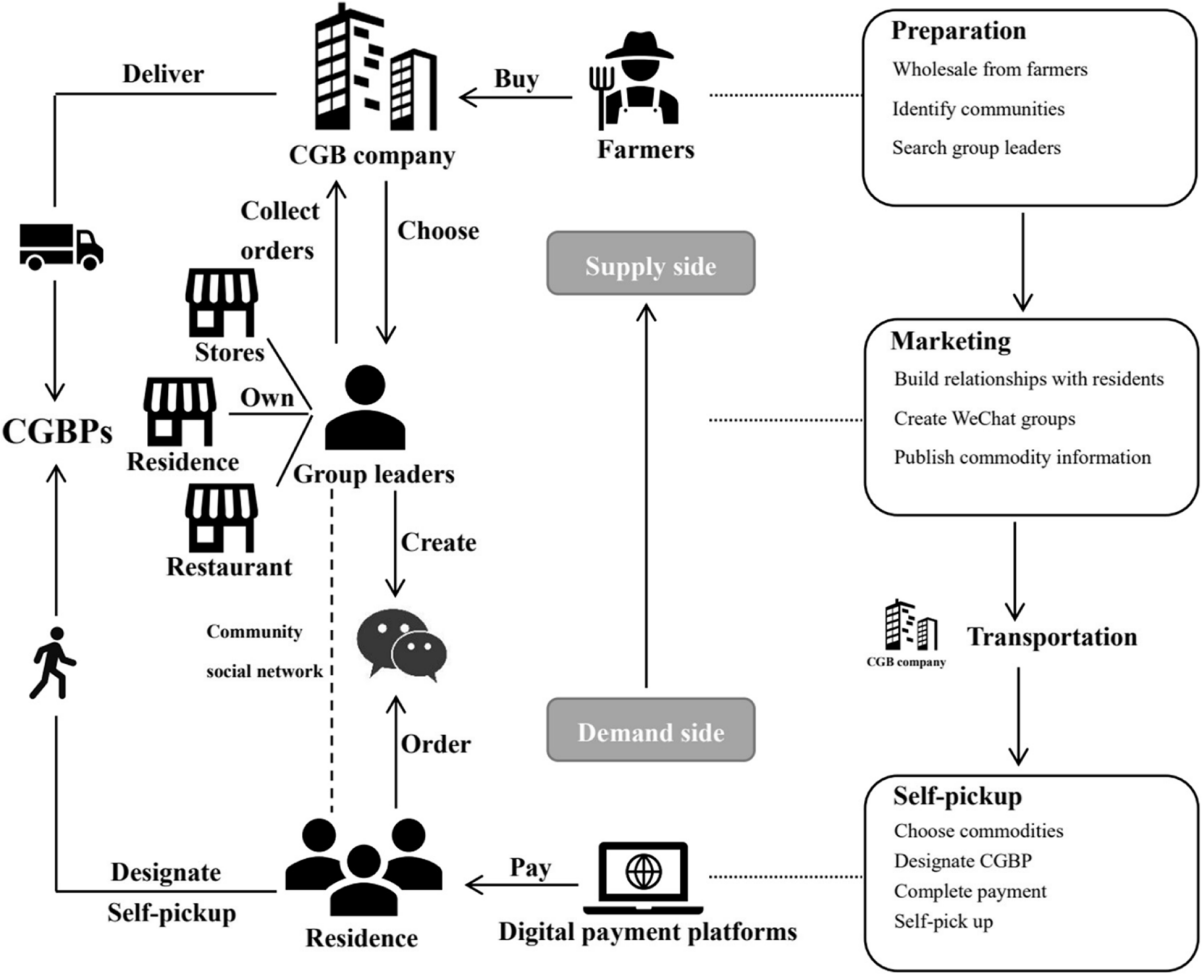
CGB operation model

The whole operation model can be summarized as: (CGB companies) Wholesale from farmers—Identify communities—Search group leaders—(Group leaders) Build relationships with residents—Create WeChat groups—Publish commodity information—(Group members) Choose commodities—Designate CGBP—Complete payment—Self-pick up (Fig. 2).

### Characteristics of CGBPs

**Fig. 3:**
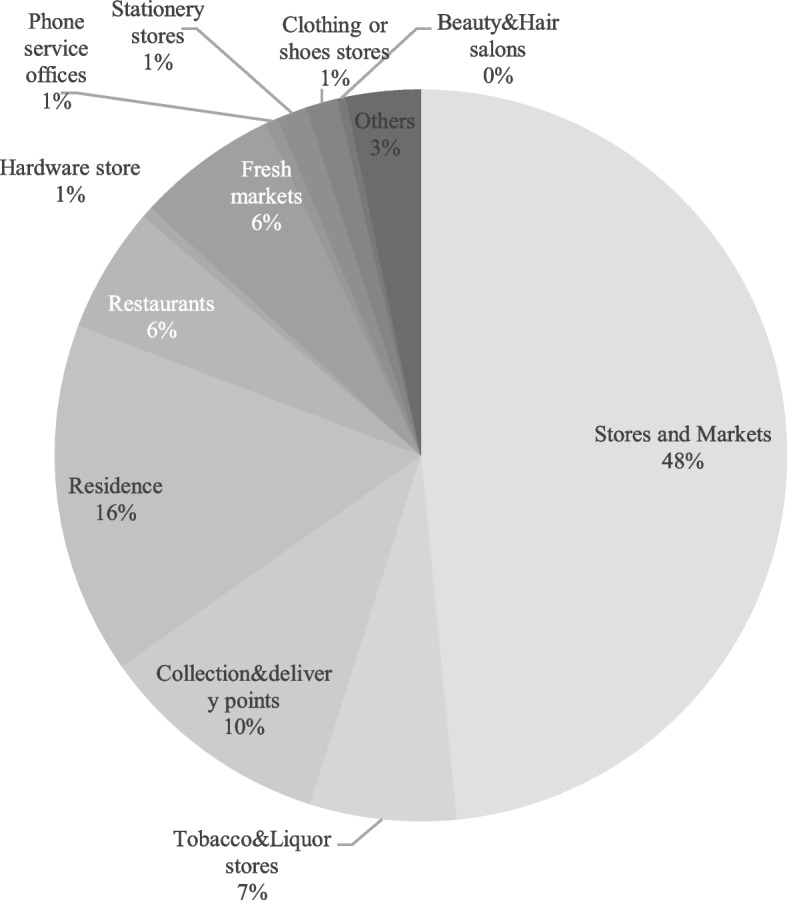
Relying on the targets of CGB

The results of the analysis of the CGB operation model showed that the CGBPs, as distribution nodes, were mainly based on stores or residences owned by the group leaders, so they were mainly joint ventures (93.7%). Categorized by relying on targets (Fig. 3), 48% of CGBP relied on stores and markets in the sinking market that interacted most frequently with community residents. CGBPs and stores or markets served primarily communities with the same location conditions. Additionally, most of the consumers of stores and markets were residents of the community, providing the growing potential customer base and interpersonal networks for the development of CGB, and CGB contributed additional traffic to stores and markets, where unplanned consumption occurred frequently. The turnover would increase significantly with increased traffic. Therefore, for mutual benefit and common development, CGB companies preferentially chose stores or markets as CGBPs.

Followed by residence and existing collection & delivery points, representing 16% and 10%, respectively. The former provided employment opportunities for unemployed people and housewives and could operate with low or no external costs. The latter improved the functions of existing commercial and state-owned self-pick-up points at a low cost. Approximately 6% of CGBP relied on tobacco & liquor stores or Fresh markets. Few CGBPs relied on clothing or shoe stores, stationery stores, and phone service offices (China Mobile, China Unicom and China Telecom), while fewer CGBPs were set up as hotels, lottery shops, and maternity shops. In summary, the CGBPs presented the characteristic of ‘convenience store-based and multitype coexistence’.

## Spatial pattern of CGBP and its influencing factors

### Spatial pattern of CGBPs

The Moran* I* index of CGBP in the study area was 0.44, greater than 0, with a significant P-value and Z-score, indicating that the distribution of CGBPs was clustered at a 99 percent significance level. The SDE analysis results indicated that: There was no obvious spatial directionality of CGBPs. The spatial layout was symmetrically distributed along the axis connecting the southwest and northeast corners of the Xi’an Circumvallation, centered around the revolution park in the Xincheng district. The regional shape and urban planning were the main reasons. The study area was approximately 31.7 km from north to south and 44.4 km from east to west, and the aspect ratio was close to 1, so the urban shape was relatively regular. Additionally, six central districts of Xi’an adopted a coordinated development planning pattern, so the gaps in economic, population and infrastructure conditions of the six districts were relatively narrow, which was conducive to a uniform distribution of CGBPs, explaining why there was no obvious spatial directionality of CGBPs. However, the axis inclined slightly because the spatial planning of the urban system of Xi’an City pointed out that the Beijing-Kunming Railway development belt along the southwest-northeast direction was one of the most important economic corridors in Xi’an. In addition, the north Yanliang-Gaoling and south Chang’an District, two subcenters of the city, had a certain influence on the distribution of CGBPs. Finally, the CGBPs showed an elliptic distribution pattern with a small oblateness.

**Fig. 4:**
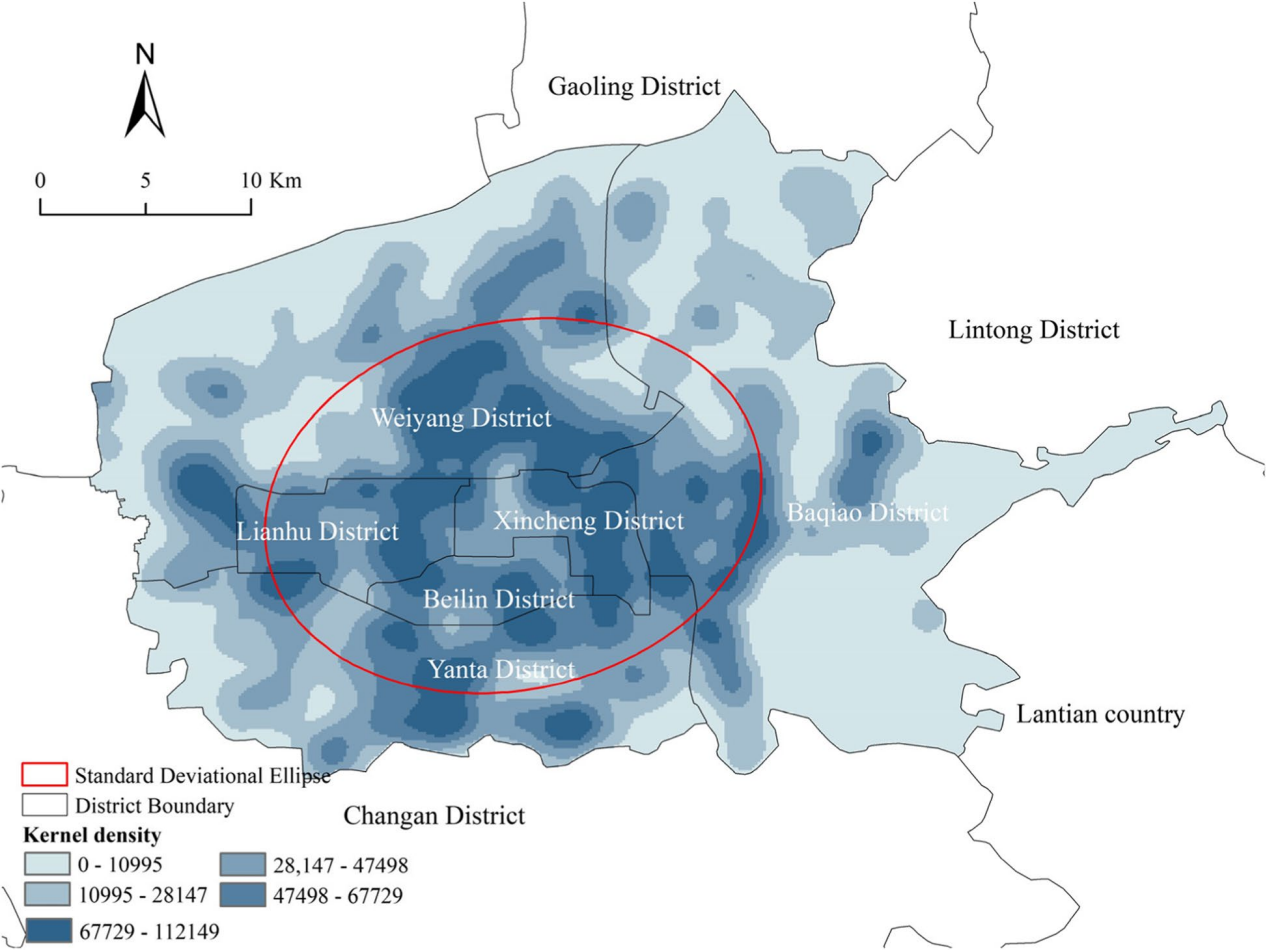
Kernel density and standard deviational ellipse of CGBPs

Furthermore, using the kernel density estimation, the results shown in Fig. 4 indicated that the density of CGBPs showed a low–high-low circular distribution pattern from the Palace of Tang Dynasty outwards. The Palace of Tang Dynasty is located in the northeast of the central area of Xi’an, east of Weiyang Road, west of Taihua Road, north of Ziqiang East Road and south of Xuanwu Road, and the cultural relics protection area is 3 ${km}^{2}$, and the construction control area is 6.5 ${km}^{2}$. Because of high land rent and strict regulations for areas near cultural heritages, the land was mainly used for historical exposition (Dahua, 1935), cultural exchange (Daming Palace National Heritage Park), commercial (Wanda Plaza) and transportation (Xi’an railway station) rather than residence. Therefore, the CGBPs were less dense and scattered at the edges. However, the CGBPs were clustered within the 2 km ring buffer of the Xi’an second ring road. The main reason was that the Second Ring Road was the urban expressway and one of the most important skeletons of the road network. Due to convenient transportation along the Second Ring Road, universities, dormitories, factories, and their staff quarters, and commercial estates tended to be built along it, providing a large number of relying on targets and potential consumers for CGB. Outside the second ring road, the density of CGBPs generally showed a downward trend, except for areas along the elevated highway and other arterial roads. The decline rate in the south was lower than that in the north because the Yanta District in the south was the district with the highest GDP and shared high-quality and rich educational resources with the Chang’an District in the south. Attracted by the developed economy and high-quality education, a large number of commercial communities were built, accompanied by dense CGBPs. Finally, the eastern part of the Baqiao district was the Xi’an International Trade & Logistics Park and the Chanba Ecological Area without suitable targets, so the density of CGBPs was the lowest.

### Potential factors influencing the spatial pattern of CGBPs

Commercial facilities around communities were the most common relying targets of CGBPs, and community residents were target customers of CGB. Therefore, it could be inferred that the spatial distribution of CGBPs coincided largely with that of communities, so the number of communities and their attributes, such as types, average price, etc. had an important impact on the location of CGBPs. Furthermore, similar to the traditional retail industry, it was predicted that CGBPs tended to be located in highly populated (Simkin, 1990) and easily accessible areas (Mazze, 1972). Furthermore, the level of development of the regional retail industry was generally determined by GDP. The higher the GDP, the greater the number and level of retailers (Vandell & Carter, 1994). To sum up, from the three dimensions of service objects, relying on facilities and economic environment, six variables were selected: the number of communities, housing type, average housing price, population density, road network density, and economic development level (GDP). Among them were four housing types: residences, office buildings, shops, and villas. The CGB market strategy focuses on price advantages, targeting middle and low-end markets, and serving price-sensitive community users (Chen, 2021), so 4 ~ 1 scores were assigned to residences, office buildings, shops, and villas in turn, indicating the similarity with the portraits of CGB’s target customers. The higher the score, the more likely residents of this type of housing would be to spend on the CGB.

**Fig. 5:**
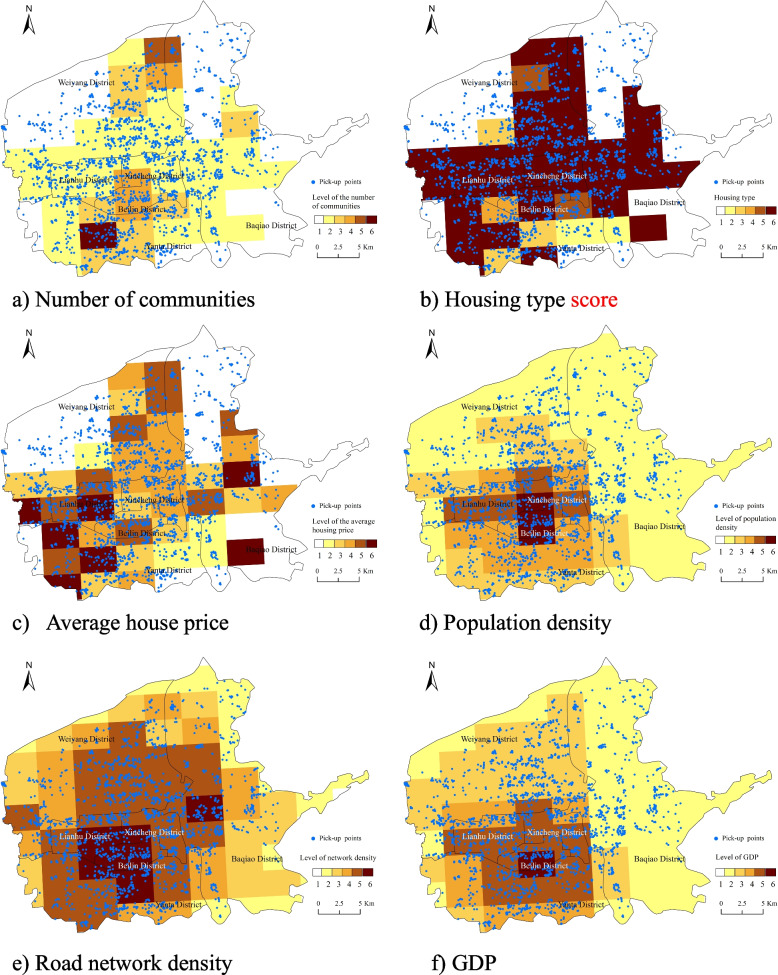
Spatial distribution map of 6 potential influencing factors

First, the study area was divided into 10 × 10 grids. Then, the number of communities, sum of housing type scores, average house price, population density, road network density and average GDP for each grid were calculated and divided into six levels using the Natural Breaks method respectively. Finally, the spatial distributions of 6 potential influencing factors were visualized on the map (Fig. 5), the level of the factors was represented by different colors. The darker the color, the higher the level.

Figure 5 showed that each influencing factor’s value was centered on Xi’an Bell Tower and peaked in Lianhu District, Xincheng District and Beilin District, with a long urban development history, and gradually decreased outward. The center of gravity was in the southwest. Except for housing type, the values of potential influencing factors in the southern region were higher than those in the northern region, while the housing type score was distributed in an inverted T shape. The Baqiao District was dominated by ecological land, so all indicators’ scores were the lowest. The results showed that the areas with a high concentration of CGBP largely overlapped with high-value areas of each potential influencing factor to different degrees. Therefore, it could be inferred that these potential influencing factors were positive driving factors for the the location choice of CGBPs.

### Results of the factor detector

To further quantitatively test the factors that influence the spatial distribution of the CGBPs in the central districts of Xi’an, this study adopted a statistical analysis method based on the perspective of spatial heterogeneity, Geodetector.

Table 2 showed that the spatial distribution of CGBPs was significantly and positively determined by the number of communities, population density, GDP (*P* < 0.01) and housing type score (*P* < 0.05). Among all factors, the explanatory power of the number of communities to the spatial distribution of CGBPs reached 86.43%, which was much higher than other factors. This was mainly because CGB was initially established based on neighborhood relationships in targeting community users. In order to expand market share and provide residents with more convenient self-pickup services, ‘one community, one point’ was the goal of the CGBPs construction. Therefore, the areas where CGBPs were highly concentrated intersected with dense community regions. The second strongest explanatory factor was the population density (q = 21.22). The number of consumers determined the market size. In order to meet the demand threshold and increase turnover, CGBPs tended to be located in highly populated areas. Furthermore, GDP was an important index to measure the economic development of a region, explaining the location of the CGBPs at the 18.86% level. The higher the GDP, the more advanced the regional commercial and service facilities (Estevão et al., 2020), which could provide a large number of suitable targets for CGBPs. Furthermore, residents living in high-GDP areas had stronger buying power (Toossi, 2002) and had greater access to the Internet (Kaya et al., 2017), so they accepted the new ‘online + offline’ retail model like CGB more easily, which encouraged CGBPs to cluster in economically developed areas of the city. In addition to the number of communities, the community type also influenced the location of CGBPs. Middle-aged and older people who are sensitive to price are the target customers of CGB (Chen et al., 2021). To shorten their travel time, the residence was the best choice for setting up CGBPs. On the one hand, CGBPs located in office buildings or stores were fewer due to high rents and limited relaying targets. On the other hand, middle- and upper-class people living in villas paid more attention to the commodity quality rather than the price, so they were less likely to buy on the CGB platform.

**Table 2:** Results of factor detector

Indictor	Statistic q statistic (%)	*P*-value
Number of communities	86.43	0.00^**^
Housing type	13.88	0.03^*^
Average house price	7.35	0.13
Population Density	21.22	0.00^**^
Traffic density in the road network	7.68	0.30
GDP	18.64	0.01^**^

*q* how much the dependent variable affects the independent variable

^*^0.01 < *P*-value ≤ 0.05, **P-value < 0.01

Unlike the traditional retail industry, road network density was not an important consideration when choosing a CGBP location. The main reason was that the service coverage divided by the community was small, so the accessibility to CGBP within service coverage was high with a slight disparity, which was weakly related to the road network. Housing prices were primarily determined by surrounding business, medical facilities, traffic conditions, and school districts. The distributions of these public resources were relatively even in six central districts, so the variance of the housing prices was also small. Therefore, it had little influence on the spatial distribution of CGBPs at the urban scale.

### Interaction detector results

The interaction detector was used to detect the interaction of two factors in explaining the spatial distribution of CGBPs. There were 15 pairs of interaction results for the 6 factors (Table 3). All of them showed either bi-enhanced (the explanatory power was greater after interaction) or nonlinear-enhanced (achieving the effect of “1 + 1 > 2”). In other words, any two independent variables had stronger explanatory power after interaction, indicating that the spatial distribution of the CGBPs was driven by multiple factors.

**Table 3:** Results of the interaction detector analysis

Interaction	Number of communities	Housing type	Average house price	Population Density	Traffic density in the road network	GDP
Number of communities	0.8643					
Housing type	0.9067	0.1388				
Average house price	0.9023	0.2424	0.0736			
Population Density	0.9263	0.2867	0.3381	0.2122		
Traffic density in the road network	0.8968	0.2024	0.2714	0.2930	0.0768	
GDP	0.9193	0.3152	0.3451	0.2903	0.2270	0.1864

The explanatory power of the interaction between the number of communities and population density was the strongest (0.9263), followed by the number of communities and GDP (0.9193), the number of communities and housing types (0.9067), the number of communities and average house price (0.9023) and the number of communities and road network density (0.8968), showing that the combination of the community number and other factors could explain the spatial distribution of CGBP to a large extent.

In summary, the spatial distribution of the CGBPs was essentially determined by the number of communities in each region and the economic and demographic conditions would work on this basis. In other words, CGBPs maintained a mutually beneficial and symbiotic relationship with the communities, and serving more communities was the primary goal of the location choice. On this basis, residential-based housing type, high population density, and great economic conditions would increase the attractiveness of an area to CGBPs. The high density of CGBPs in the Beilin, Xincheng, and Lianhu Districts proved this point.

## Accessibility evaluation and location optimization of CGBPs in the post-epidemic era

### Accessibility to CGBP

With the continuous expansion of urban boundaries and the development of multicentralized cities, the concept of a “community living circle” has been proposed. It takes people’s walking time as the standard for grading facilities, helping residents meet the corresponding living needs within a suitable walking time to guide the rational layout of supporting facilities (Liu & Chai, 2015). In the post-epidemic era, with the removal of control measures including lock-downs, the residential communities were no longer self-managed and self-organized. The “community living circle” once again replaced the physical boundaries of the community and better reflected the real daily activity space for residents. CGBPs are an important part of the community living circle as support facilities for community life.

Buffer and network analysis were used to identify the service coverage of CGBPs. According to the Urban Residential Area Planning and Design Standards (Table 4), 300, 500 and 1000 m from the CGBP were taken as distance impedance, and 5, 10, and 15 min were taken as time impedance, corresponding to the 5, 10 and 15 min community living circle, respectively. The average walking speed was set as 1.4 m s—1 (Feng & Yang, 2015).

**Table 4:** Different categories of living circles

Classification Criteria	5-min living circle	10-min living circle	15 min living circle
Walking distance (m)	300	500	800 ~ 1000
Residential population (person)	5000 ~ 12,000	15,000 ~ 25,000	50,000 ~ 100,000
Number of dwellings (sets)	1500 ~ 4000	5000 ~ 8000	17,000 ~ 32,000

**Fig. 6:**
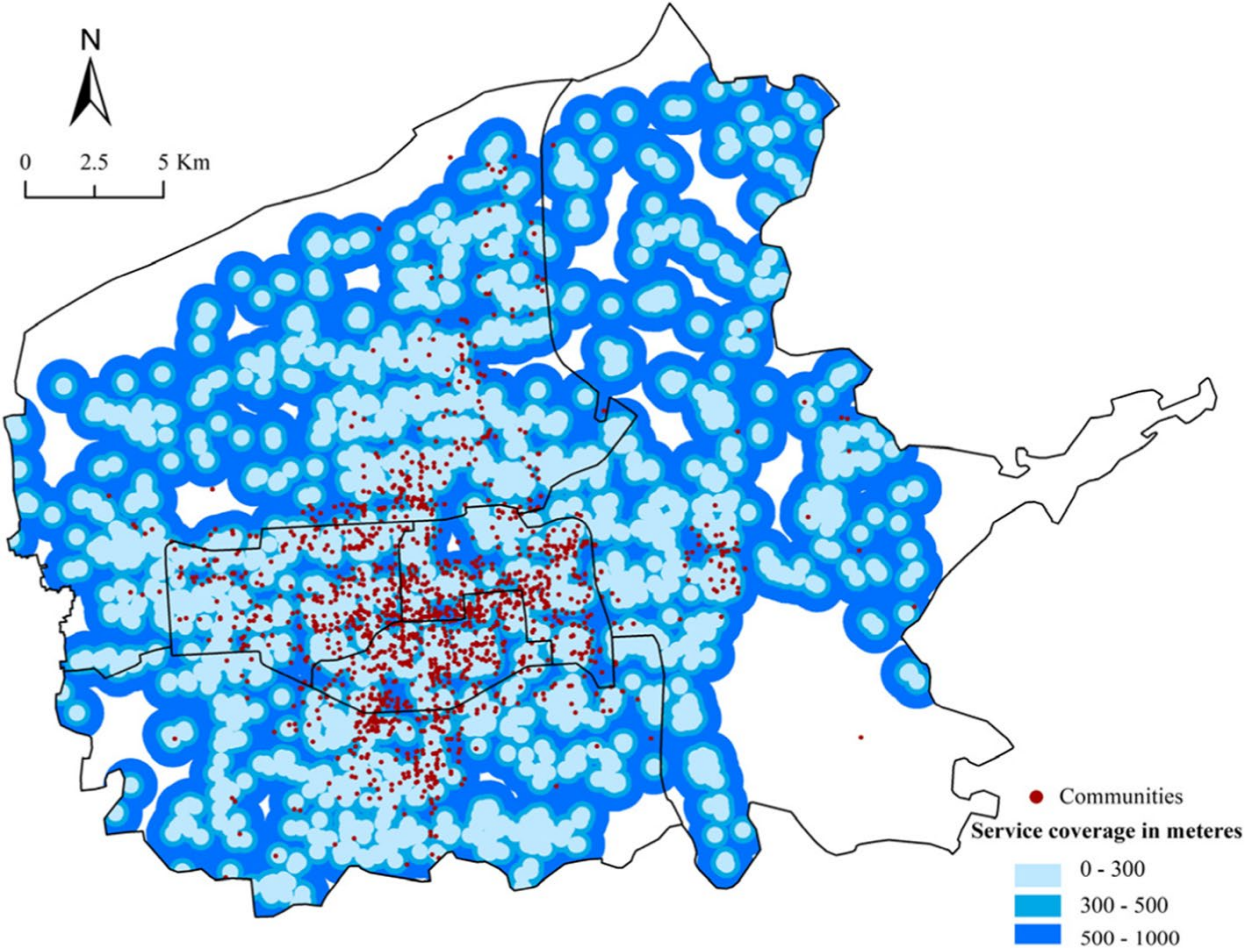
Various accessibility modes in terms of service coverage area by distance

**Fig. 7:**
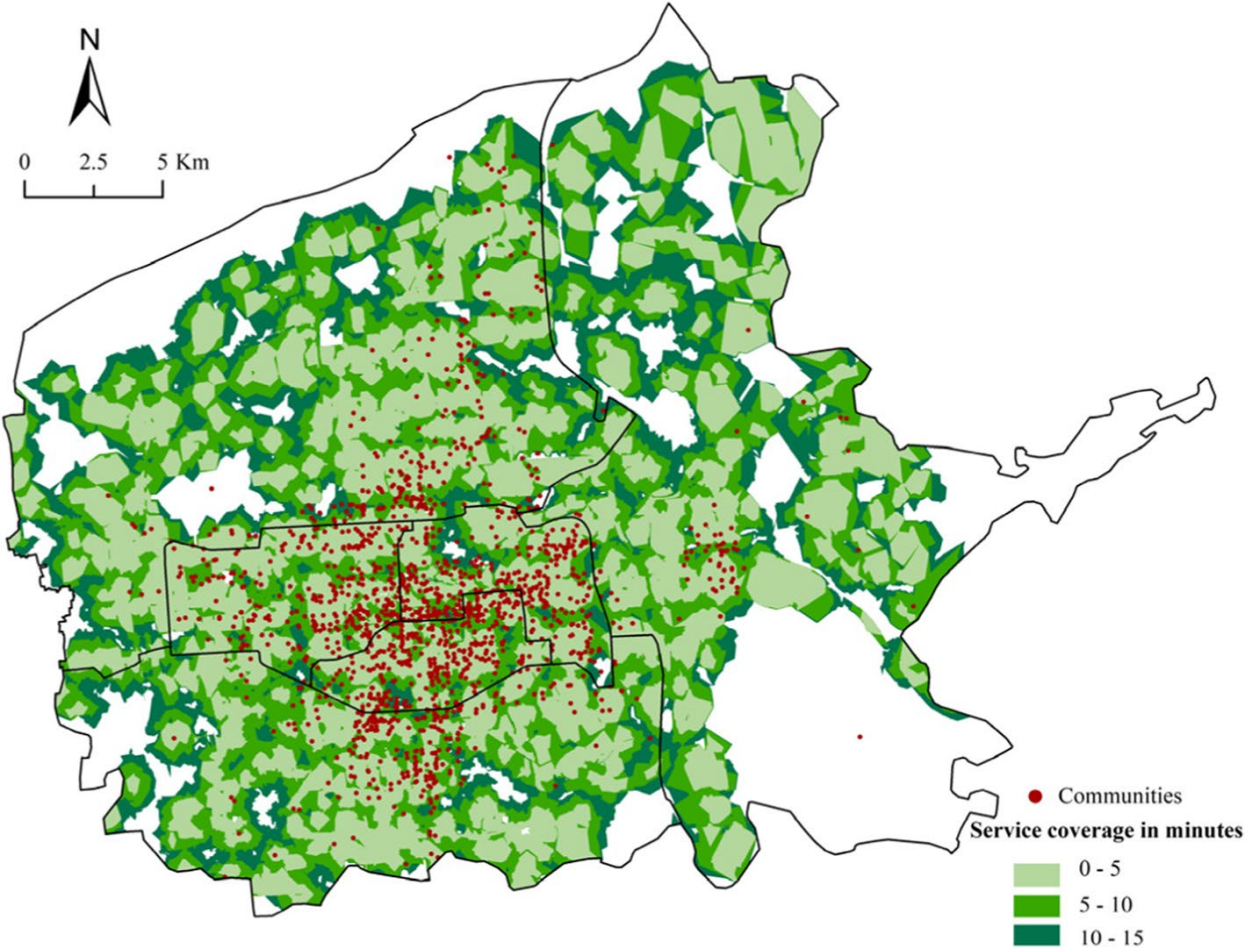
Various accessibility modes in terms of service coverage area by time

Figures 6 and 7 revealed the ranges for three walking-accessibility modes by colors based on the “community life circle” theory: 5 min (300 m), 10 min (500 m) and 15 min (1,000 m). The darker the color, the lower the CGBPs’ accessibility. Table 5 showed 66.45% of the communities had a less-than-300 m straight-line distance from CGBPs and 67.97% could walk to CGBPs in 5 min, while only 0.11% of the communities had no access to CGBPs within 1,000 m, and 0.94% took more than 15 min to walk there.

**Table 5:** Quantitative results of accessibility to CGBPs based on the theory of the community life circle

Community life circle	Accessibility	Standard	Distance/Time	Number of communities in living cycles	Percentage (%)
5-min living circle	High	Distance	300 m	1834	66.45
		Time	5 min	1876	67.97
10-min living circle	Medium	Distance	500 m	2467	89.38
		Time	10 min	2590	93.84
15-min living circle	Low	Distance	1000 m	2757	99.89
		Time	15 min	2734	99.06

In summary, in the 5-min community living circle, more than 1,800 communities were covered by CGBPs, more than 65% of the total number, and almost all communities were accessible to CGBP in the 15-min community living circle.

High access areas to CGBP (5-min community living circle) covered more than 90% of the area in Xincheng, Lianhu and Beilin districts, as well as the central part of Yanta district, southeast of the Weiyang district, and west of the Baqiao district. Low-accessibility areas to CGBP (outside the 15-min community living circle) were mainly distributed in: 1) Villages on the north side of Chang’an City Site in the west of Weiyang District. Due to its proximity to the Weiyang Palace in Chang’an City of Western Han dynasty, most neighboring villages had been relocated to protect cultural relics, and only some remote villages were reserved. The number of residents had decreased significantly and the demand threshold for CGB could not be met. 2) The Huashan plant communities in the north of the Weiyang district. These communities were affiliated with China Northwest Weapon Manufacturing Corporation. Most residents were retired soldiers who were too old to have access to the Internet, making online shopping difficult. Furthermore, stores in the community were factory-owned. To protect their own interests, they were unlikely to cooperate with other companies. 3) Villages like Zhaizi Village, Zhongyun Village, and Mi Village in the northeast of Baqiao District. This area was mainly for agricultural production and Bailuyuan tourism industry. Fresh products could be self-sufficient and exported rather than purchased from outside. 4) Around the Chengxi Bus Station in Lianhu District and Xi’an Railway Station in Xincheng District. The land rent around transportation hubs was relatively high, mainly used for commercial land with visitors as the main consumer group. The fresh market was small. 5) Around the northwest wholesale market for agricultural products in Weiyang District. The wholesale market was one of the main competitors of CGB, with advantages of large scale and low prices. It could meet the needs of surrounding residents for fresh products and squeeze the market for CGB.

### Location optimization of CGBPs

#### Model design

The accessibility analysis above revealed that CGB could reach more residents in fewer numbers by optimizing CGBPs’ locations. There were 2,443 CGBPs in the study area, with an average of 0.88 CGBP for each community. However, Table 6 showed a huge disparity in the average CGBP number owned by each community between different districts. Although there were dense CGBPs in the Lianhu, Xincheng, and Beilin districts, the average CGBP number for each community was less than 1 due to the higher community density, while in the Yanta district, Weiyang district and Baqiao district, each community had more than 1 CGBP on average due to the relatively lower density and sparser community distribution. Among all districts, the communities in Baqiao District had the highest average CGBP number, reaching 4.21.

**Table 6:** The average number of CGBPs for each community in different districts

District	The number of communities	The number of CGBPs	The average number of CGBPs for each community
Lianhu	383	225	0.59
Xincheng	664	186	0.28
Beilin	601	120	0.20
Yanta	502	585	1.17
Weiyang	477	757	1.59
Baqiao	133	560	4.21

Based on this, the following aspects should be considered when optimizing the location of CGBPs: 1) More CGBPs should be built to relieve the pressure on existing points in places where multiple communities share a single CGBP, such as the Lianhu, Xincheng, and Beilin districts. 2) The spatial distribution of CGBPs within Yanta and Weiyang districts is uneven, so some CGBPs should be relocated within the district. 3) The Baqiao district is dominated by agriculture and tourism, with a low demand for daily fresh products. The CGBP number currently exceeds the demand of local residents. To avoid waste of resources, some CGBPs should be replaced or removed.

Therefore, a combination of the minimize facilities model and the maximum attendance model was applied in this research to optimize the location of CGBPs.

First, according to CGBPs’ relying targets and location requirements (close to the community and easily accessible): 927 well-located convenience stores, restaurants, collection and delivery points, and barber shops were chosen as candidates (meeting the conditions of being less than 50 m from the nearest community entrance while being distributed along the street). Together with the existing 2,433 CGBP, a total of 3,360 points were chosen as alternative locations. To maximize market share, 57 large supermarkets, new retail stores and fresh markets were chosen as competitor facilities. 2,760 communities were chosen as demand points. Second, the impedance was set as the travel time and the impedance interrupt was set as 5 min. Then, the power function was applied as an impedance condition and the impedance parameter as well as the power index were set to 2, calculated based on the target customers, consumer spending, repurchase frequency, product category and business data of the CGB using ArcGIS Business Analyst. The set conditions meant that, on the premise that the CGBPs could be reached within 5 min on foot, the probability that a resident choosing a CGBP would decrease by the square of the distance between the community and the CGBPs.

To explore the minimum number of CGBPs to meet the needs of the residents in the study area, the minimize facilities model was applied. The result showed that at least 472 CGBPs should be built. Based on this, maximize attendance model was further used to achieve the goal of maximizing facility efficiency. The “facilities to choose” was increased in steps of 10 from the minimum number (472) until the additional CGBPs contributed less than 0.1% to the 5-min service coverage rate.

#### Optimization results

**Fig. 8:**
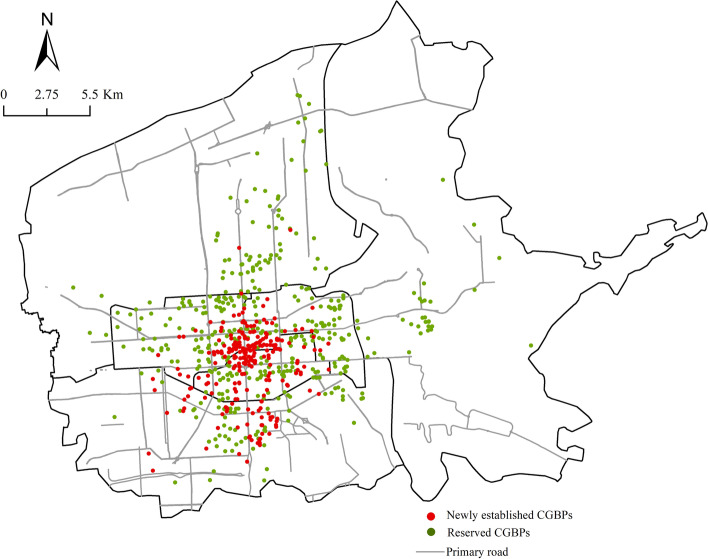
Location Optimization of CGBPs

Figure 8 showed that finally 642 locations were selected to establish CGBP, which could serve 76.67% of communities in the 5-min community living circle in six central districts of Xi’an. Among them, 248 new CGBPs were added, mainly cafes, convenience stores and hotels at the junction of Xincheng, Lianhu, and Beilin Districts and the northern part of Yanta District. Most CGBPs in downtown and the middle of Yanta district were preserved, while in Weiyang district and Baqiao district, it was suggested to cancel some existing CGBPs and replace them with farmers’ markets, mobile vendors, and supermarkets, which were not only conducive to the development of local agricultural economy and protection of farmers’ rights and interests but also avoided wasting resources in decentralized communities. The spatial coupling between CGBPs and communities was higher after location optimization. With the Xi’an Bell Tower as the center, the optimized CGBPs were distributed in the shape of ‘ + ’, concentrated on the north–south and east–west central axes of the Xincheng, Lianhu, and Beilin districts. Each CGBP served up to 4 communities, greatly improving service efficiency.

#### Sensitivity analysis

Three parameters $N$, $v$ and $t$ were chosen to conduct local sensitivity analysis by the factor transform method. 10% and 20% of three parameters were increased/decreased respectively, while other parameters were kept constant.

$${s}_{ji}=\frac{\partial {F}_{j}(X)}{\partial {x}_{i}}=\frac{\partial {F}_{j}(X)/{F}_{j}(X)}{\partial {x}_{i}/{x}_{i}}=\frac{\partial {F}_{j}(X)}{\delta }\frac{1}{{F}_{j}(X)}$$

${s}_{ji}$ is the sensitivity coefficient of the function ${F}_{j}(X)$ to the variable ${x}_{i}$; $\delta$ is the parameter variation; Since ${F}_{j}(X)$ is a constant value, the sensitivity coefficients can be measured by the ratio of the changes in the number of CGBPs after the optimization to parameter variations. Therefore, the changes in the number of CGBPs after optimization was used as the output (Table 7).

**Table 7:** Changes in the number of CGBPs under different parameter conditions

Parameter	Changes in the number of CGBPs (%)				
	-20%	-10%	0	10%	20%
$N$	-0.68	-0.43	0	0.79	2.13
$v$	0.58	0.29	0	-0.29	-0.59
$t$	0.58	0.29	0	-0.29	-0.59

The number of CGBPs is negatively correlated with the impedance cutoff $t$ and the speed of walking $v$ while positively correlated with the power index $N$. Among the parameters analyzed, power index $N$ has the highest sensitivity and greatest effect on this optimization model. When this parameter increases by 20%, the variation in the number of CGBPs accounts for 2.13%.

## Discussion and conclusion

### Discussion

On January 26, the National Health Commission announced that novel coronavirus infection would be adjusted from “Class B A” to “Class B B” on January 8, 2023, which heralds China’s full entry into the post-epidemic era. With the removal of lockdown measures, both increasing customer demand for product quality and growing purchasing channels for consumers (traditional retailers, community fresh food stores and fresh food e-commerce) have squeezed CGB’s market, undermining their advantage of being located within the community. However, it is predicted that it still has certain advantages and space for development in the post-pandemic era, and will continue playing an important role in the community living circle due to the following reasons:

1. CGB adopts an innovative business model which combines the advantages of “online + offline e-commerce model” (Tsai et al., 2015) and “pre-sale + group buying business model” (Zhu et al., 2022; Posselt & Gerstner, 2005). The former reduces costs and transportation losses by self-built logistics network and self-pickup points. The latter helps CGB basically achieve “zero inventory”.
2. The CGB system centralizes procurement and distribution to reduce the intermediate circulation price. The cost saved is directly returned to consumers by decreasing the commodity price.
3. The CGB has led to an extremely high repurchase rate. On the one hand, the group was created by acquaintances like housewives, convenience store owners in the community. They build trust face-to-face with potential customers and stay in touch with them. On the other hand, fresh products as a daily rigid demand account for nearly half of CGB goods.

In summary, low prices, convenience, flexible cash flow, zero inventory, and regular customers have ensured CGBP’s survival before, during, and after the pandemic. Moreover, because of the continued impact of the epidemic on people’s lifestyle and consumption habits, it can be forecasted that the importance of CGBPs during the pandemic will maintain towards the post-pandemic phase. However, some adjustments can be made to help CGBPs better adapt to the post-epidemic era. For example, without the boundary limitation of closed-management communities, the connection between CGB’s supply chain and other new retail logistics systems can be built to save logistics resources and shorten delivery time. Furthermore, the spatial integration of CGBPs from various CGB brands should be considered in the future.

## Conclusion

The findings suggested that the CGB operation model could be divided into preparation (wholesaling commodities, determining communities, choosing group leaders, creating Wechat groups and setting up CGBPs), marketing (publishing commodities’ information and paying), transportation (collecting orders and delivering) and self-pickup. The CGBPs that played an important role in the final link mostly existed in the form of joint ventures (93.7%) and due to complementary resources, nearly half of them relied on stores and markets in the sinking market that interacted most frequently with the community residents. The spatial distribution of the CGBPs was concentrated in the central parts of Xi’an. Affected by urban planning, land use and cultural relics protection regulations, they showed an elliptic distribution pattern with a small oblateness, and the density showed a low–high-low circular distribution pattern from the Palace of Tang Dynasty outwards. Furthermore, the number of communities, population density, GDP, and housing type were important driving factors of their spatial pattern. The explanatory power of the interaction between the community number and other factors was greater than 89%, demonstrating that the number of communities had a critical influence on CGBPs’ spatial distribution. In terms of accessibility to and service coverage of CGBPs, more than 90% of the communities in Xincheng, Lianhu, and Beilin districts had access to CGBPs in the 5-min community living circle both in time and distance, but the average number of CGBPs for each community was less than that in other administrative districts. To avoid wasting resources and improve facilities’ efficiency, it was suggested to add 248 CGBPs at the junction of Xincheng, Lianhu and Beilin districts and the northern part of Yanta district, and retain 394 CGBPs around Bell Tower and the central part of Yanta district, and replace the remaining CGBPs with farmers’ markets, mobile vendors, and supermarkets in Weiyang District and Baqiao District.

Due to technical issues and data accessibility, there is still room for optimization. First, in terms of the study area, only CGBPs data in 6 central districts were collected. Suburbs and counties under the jurisdiction of Xi’an were not included, so only CGBPs’ spatial pattern in urban areas was shown. The differences in patterns between urban and rural areas were ignored. Second, the spatial pattern of CGBPs was the result of a combination of multiple factors. Considering the difficulty of data acquisition, some socio-demographic factors, such as Internet penetration, the size of mobile payment users and age structure of each community, were not considered in the analysis of factors influencing CGBP location selection. Third, the research object involves more than 2,000 points, but the differences in their service capability, number of fans and organization form etc. have not been considered when conducting accessibility evaluation because of the limited data. Finally, more variables such as travel preference, neighborhood closeness, price sensitivity, etc. are expected to be added in the optimization model to make it more similar to the real life.

A spatial analysis and optimization study on CGBPs helps to improve the final link of the CGB supply chain and the urban planning of the community’s living circle. In particular, this study investigated the influencing factors, accessibility and location optimization of the CGBPs through multiple methods and models. The findings contributed to promoting the integration of resources, developing effective strategies regarding the CGBP location planning, and achieving sustainable development of CGB companies.

## Data Availability

All the data used for several analyses are freely available and the resources are mentioned within the paper.
